# Supplementary material for: Vitamin D Modified DSS-Induced Colitis in Mice via STING Signaling Pathway
Source: Biology (Basel). 2025 Jun 18;14(6):715. doi: 10.3390/biology14060715 (PMC12190092; doi:10.3390/biology14060715)
Supplement: Supplementary file 1 [file biology-14-00715-s001.zip › Supplementary Materials S1: 16s-report of gut microbiota/Groups/taxanomyBar/taxon_hist_group/include_all/top10/Phylum.distribution.pdf]

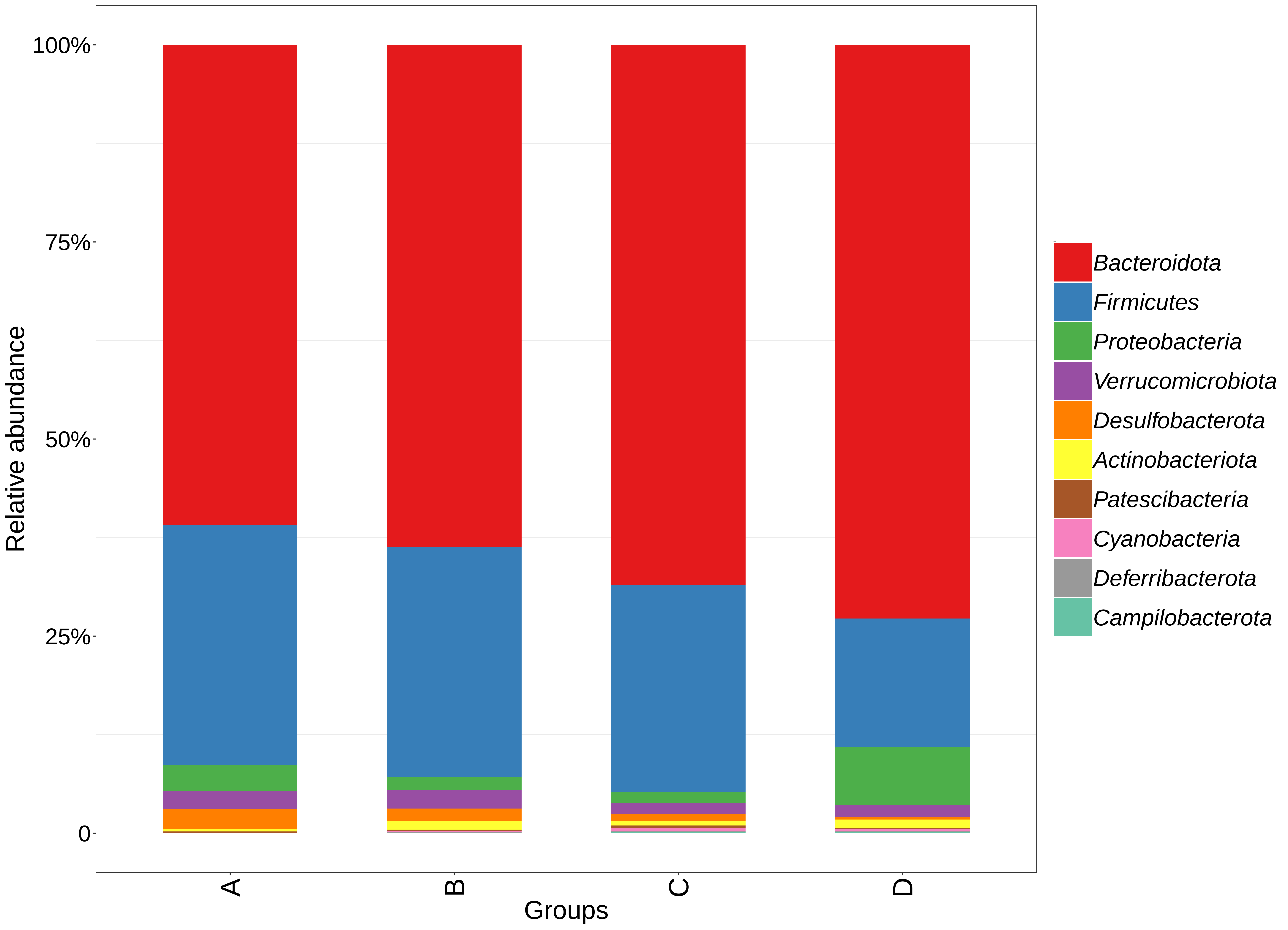

100%
75%
50%
25%
0
Relative abundance
A
B
C
D
Groups
Bacteroidota
Firmicutes
Proteobacteria
Verrucomicrobiota
Desulfobacterota
Actinobacteriota
Patescibacteria
Cyanobacteria
Deferribacterota
Campilobacterota
